# Porcine Blood and Liver as Sporadic Sources of Hepatitis E Virus (HEV) in the Production Chain of Offal-Derived Foodstuffs in Poland

**DOI:** 10.1007/s12560-021-09475-z

**Published:** 2021-04-23

**Authors:** E. Bigoraj, W. Paszkiewicz, A. Rzeżutka

**Affiliations:** 1 Department of Food and Environmental Virology, National Veterinary Research Institute, Al. Partyzantów 57, 24-100 Puławy, Poland; 2 Department of Food Hygiene of Animal Origin, Faculty of Veterinary Medicine, University of Life Sciences in Lublin, ul. Akademicka 12, 20-950 Lublin, Poland

**Keywords:** Hepatitis E virus, Porcine adenovirus, Detection, Blood for human consumption, Pig’s liver

## Abstract

Pig’s blood and liver are valuable edible slaughter by-products which are also the major ingredients of offal-derived foodstuffs. The aim of the study was an evaluation of the occurrence of hepatitis E virus (HEV) and porcine adenovirus (pAdV) as an index virus of faecal contamination in pig’s blood and liver for human consumption. In total, 246 samples of retail liver (*n* = 100) and pooled pig’s blood (*n *= 146) were analysed for the presence of HEV and pAdV. Blood samples were individually collected from 1432 pigs at slaughter age. Viral genomic material, including RNA of a sample process control virus was isolated from food samples using a QIAamp® Viral RNA Mini Kit. Virus-specific IAC-controlled real-time PCR methods were used for detection of target viruses. HEV RNA was found in 6 (2.4%; 95% CI: 0.9–5.2) out of 246 samples of tested foodstuffs. The virus was detected in pig’s blood (3.4%; 95% CI: 1.1–7.8) and liver (1.0%; 95% CI: 0.0–5.0) with no significant differences observed in the frequency of its occurrence between the two by-products (*t* = 1.33; *p* = 0.182 > 0.05); however PAdV was detected more frequently in pig’s blood than in liver (*t* = 4.65; *p* = 0.000 < 0.05). The HEV strains belonged to the 3f and 3e subtype groups and the pAdV strains were assigned to serotype 5. PAdV was detected in pigs regardless of the farm size from which they originated. The number of animals raised on the farm (the farm size) had no influence on the occurrence of HEV or pAdV infections in pigs (*F* = 0.81, *p* = 0.447 > 0.05 for HEV; *F* = 0.42, *p* = 0.655 > 0.05 for pAdV). Although HEV was detected in pig’s offal only sporadically, consumers cannot treat its occurrence with disregard as it demonstrates that HEV-contaminated pig tissues can enter the food chain.

## Introduction

Foodborne transmission of hepatitis E virus (HEV) to humans was described for the first time in 2003 and infection was linked to consumption of raw deer meat (Tei et al., 2003). In the subsequent years, cases of HEV infections were linked with ingestion of pig liver–derived foodstuffs (Colson et al., 2010; Kubacki et al., 2017; Renou et al., 2014; Yapa et al., 2016), undercooked pig’s liver (Guillois et al., 2016; Miyashita et al., 2012; Yapa et al., 2016; Yazaki et al., 2003), pork (Miyashita et al., 2012) and the liver and meat of wild boars (Masuda et al., 2005; Matsuda et al., 2003; Tamada et al., 2004). Other routes of infections are consumption of contaminated water (King et al., 2020), blood transfusion (Boxall et al., 2006; Matsubayashi et al., 2004; Mitsui et al., 2004; Satake et al., 2017), organ transplantation (Kamar et al., 2008) and likely through vertical transmission from infected mother to foetus (Anty et al., 2012).

Annually, 20 million new HEV infections in humans are recorded worldwide, and in over three million individuals the infection results in acute hepatitis, while about 44 thousand affected persons die (WHO, 2019). In Europe, between 2005 and 2015, a ten-fold increase in the number of HEV infections in humans has been observed, most of which could result from foodborne transmission (ECDC, 2017). Disease in humans is caused by HEV strains classified to gt 1–4 (Milojević et al., 2019; Pérez-Gracia et al., 2015; Smith et al., 2014; WHO, 2020; Yugo et al., 2013) with 23 subtypes (3a-m, ra, 4a-i) described so far (Smith et al., 2020). Besides humans gt 3 and 4 virus strains can also infect several species of farm animals and wildlife (Smith et al., 2014; WHO, 2020; Milojević et al., 2019). It is noteworthy that the presence of anti-HEV antibodies was found more often in people eating pork liver sausage, pâté and wild boar meat (Mansuy et al., 2016). This is a consequence of the presence of the virus in tissues of asymptomatically infected animals entering the food chain, instances of which are the detection of HEV RNA in muscles (Di Bartolo et al., 2012; Garcia et al., 2020; Intharasongkroh et al., 2016), liver (Boxman et al., 2019; Feurer et al., 2018; Garcia et al., 2020), kidney and heart (Garcia et al., 2020) as well as in pig’s blood (Boxman et al., 2017; Geng et al., 2019). Food law regulations define pig’s blood and liver as meat, classifying them as offal (Regulation (EC) No. 853/2004). They are valuable edible slaughter by-products suitable for human consumption (Banach et al., 2011). Edible blood (in Poland obtained only from pigs and cattle) should be collected according to specific sanitary provisions and meet sensory, physicochemical and microbiological requirements. They include the absence of pathogenic microorganisms, in particular *Salmonella spp.*, coagulase-positive staphylococci, *Clostridium botulinum*, *Clostridium perfringens* and *Erysipelothrix rhusiopathiae* (Polish Standard PN-A-85701, 1964). For pig’s livers, the detailed organoleptic standards, the requirements for their examination, packaging and storage and the microbial recommendations are specified elsewhere (Polish Standard PN-A-82000, 1965, Polish Standard PN-A-82004, 1986, Regulation (EC) No. 853/2004, 2004, ICSMF, 1986; ICSMF, 2011).

The aim of the study was an evaluation of the occurrence of HEV and pAdV (the latter as an index virus of pig faecal contamination) in edible pig’s blood and liver used for production of offal-derived foodstuffs. In addition, virus prevalence in these food ingredients was analysed considering the size of the farm which slaughtered animals originated from, farm husbandry and feeding systems.

## Materials and Methods

### Sample Process Control Virus (SPCV)

Feline calicivirus (FCV) with a titre of 10^7.1^ TCID_50_/ml was used as a sample process control virus (SPCV) added to liver and blood samples before analysis. It belongs to the virus collection of the Department of Food and Environmental Virology at the National Veterinary Research Institute in Poland. Preparation of the virus suspension in cell culture was previously described by Rzeżutka et al. (2008).

### Samples of Edible Blood and Liver

In total, 246 samples of liver (*n* = 100) and pooled pig’s blood (*n* = 146) were analysed for the presence of HEV and pAdV. The liver was purchased through regular retailers and originated from different production batches which were sold during the years 2016–2017, while the blood was collected during multiple visits to two slaughterhouses during 2018 and 2019. The blood came from animals aged from 3 months (porkers) to 24 months (backfatter sows). Finishers aged 5–6 months constituted 99.7% of the sampled pigs. The animals came from farms of different sizes breeding fewer than 25 pigs in the case of the small farms, between 25 and 50 pigs if the breeder was medium-sized and more than 50 for the large operations. Farms were located in Mazovia, Kujawy-Pomerania, Lubelskie, Łódzkie and Podlaskie, which are central and eastern voivodeships of Poland. Characteristics of the farms and husbandry details in relation to the age and weight of slaughtered pigs and the scheme of sampling are presented in Table 1. From each slaughter batch of pigs representing a particular farm, 10 randomly selected animals were sampled. However, the number of collected blood samples depended on the number of pigs raised on the particular farm. In the case of the small farms, a single sample of pooled blood per farm was prepared for analysis consisting of individual blood samples obtained from 10 pigs raised there, with the exception of nine small farms where the number of kept pigs was less than 10 and the pooled sample consisted of blood collected from between 5 and 9 slaughtered animals. At least eight individual pigs’ blood samples were included in three pools representing medium farms. If the animals originated from the medium-sized or large farms then two and three pooled samples were analysed respectively. With this scheme, sampling resulted in collection of blood from 1432 pigs. Briefly, an individual blood sample of ~ 50 ml was collected from an incision of the major blood vessels in the neck and thoracic inlet. Blood samples were pooled without using an anticoagulant and then a tested portion of the blood of no less than 40 ml was prepared. Samples were frozen at − 20° C until use. The slaughter of the animals respected the EU animal protection legislation (Council Regulation (EC) No. 1099/2009).

**Table 1:** Characteristics of animal farms, number of slaughtered pigs and collected blood samples

Farm size	Pig			Feeding type	Housing system	No. of farms	No. of pooled blood samples	No. of animals per slaughter batch (Median)
	Age (months)	Weight (kg)	Origin					
Small	3–24	35–300	Own breeding	Own feed	Straw bedding	21	21	5–13 (9)
Medium	5–24	100–200	Own breeding	Own feed	Straw bedding	7	14	16–24 (20)
Large	5–6	105–140	Own breeding	Own feed	Straw bedding, Slatted floor	7	21	25–139 (50)
				Commercial feed		14	42	30–166 (55)
			Import	Own feed	Straw bedding, Slatted floor	4	12	168–270 (172)
				Commercial feed		12	36	39–660 (168)

Samples of pig’s liver (~ 100 g) were purchased from two local butcher’s shops in Puławy, Poland selling an assortment of meats. They were supplied with pig’s liver by slaughterhouses operating in Lubelskie province. Throughout the sampling period, each shop was visited at least twice a month. The peripheral part of the right liver lobe *(lobus hepatis dexter)* or caudate lobe *(lobus hepatitis caudatus)* was sampled. Samples were frozen at − 20 °C until use.

### Isolation of Viral RNA and Estimation of SPCV Recovery from Liver Samples

A 2 g pieces of pig’s liver were cut from defrosted organs and placed to sterile 50 ml falcon tubes. Subsequently, FCV inoculum (10 µl) was directly placed on the sample. After virus adsorption a 1 ml of PBS was added followed by a mechanical tissue disruption (Omni TH 220-PCRH, Omni International, USA). The obtained homogenate was centrifuged at 14,000 × *g* for 5 min. at 2–8 °C. Isolation of viral RNA from 100 μl of liver homogenate or frozen whole blood was performed using the QIAamp® Viral RNA Mini Kit (Qiagen, Germany) according to the manufacturer’s instructions. Nucleic acids were suspended in RNA- and DNA-free water in a final volume of 100 µl. RNA was directly used for testing or stored at − 80 °C. To confirm correct sample processing and assess the efficiency of virus recovery from each analysed liver and blood sample, an SPCV was used. The virus recovery was assessed based on a comparison of the Cq (quantification cycle) values obtained for the SPCV after sample processing with those generated for isolated viral RNA derived from the virus inoculum used for sample spiking (Diez-Valcarce et al., 2012). The results of virus extraction efficiency were classified as poor (extraction efficiency <1%), acceptable (1–10%), good (>10–50%) and very good (>50%). In addition to the SPCV, a negative control was included (reagents were used for nucleic acid isolation and water in the place of the tested sample).

### Virus Detection and Identification of HEV Subtypes and pAdV Serotypes

Detection of HEV and FCV RNAs was conducted using respectively a duplex real-time RT-qPCR (Maunula et al., 2013) containing an internal amplification control (IAC) (Diez-Valcarce et al., 2011) and a real-time RTPCR (Lowther et al., 2008). Detection of pAdV DNA was also performed by the real-time PCR method (Maunula et al., 2013). For amplification of virus nucleic acids an UltraSense™ One-Step Quantitative RT-PCR kit (Invitrogen) and a TaqMan Universal PCR Master Mix (Applied Biosystems) were employed. The reactions were conducted according to the protocols previously described (Bigoraj et al., 2020; Maunula et al., 2013). A positive reaction control consisting of plasmid DNA (pCR2.1 TOPO-rSTD) with an insertion of a cDNA (HEV) or DNA (pAdV) fragments was set up for each batch of tested samples (Martínez-Martínez et al., 2011). A reaction mixture containing water instead of the tested sample served as the negative control. The number of viral genome copies (G.C.) present in liver and blood samples was determined based on a standard curve prepared using a transcript serially diluted from 10^1^ to 10^5^ G.C./µl of HEV RNA of known concentration. It was obtained during transcription of the plasmid DNA (pDrive Cloning Vector) containing a 70 bp fragment of the ORF3 genome of HEV gt3 pig strain. The HEV genome copy number present in samples was determined by HEV RT-qPCR in duplicate reactions (Kozyra et al., 2020).

For identification of the HEV subtype or pAdV serotype of detected virus strains in edible blood and liver the ORF2 fragment (Huang et al., 2002) and fragment of the virus protein hexon gene (Wyn-Jones et al., 2011) were amplified, respectively. The resulting amplification products were separated on agarose gel and subsequently purified using the QIAquick® Gel Extraction Kit (Qiagen, Germany) according to the manufacturer’s instructions. DNA fragments were sequenced and the sequences were assembled into a consensus sequence (contig) using the Chromas program (Technelysium Pty Ltd, Australia) and analyzed using the HEV Genotyping Tool (http://www.rivm.nl/mpf/typingtool/hev). The identification of pAdV serotypes was performed based on their sequence alignment with the reference sequences of human and animal adenoviruses representing different serotypes available in GenBank. Analyses were performed using BLAST (https://blast.ncbi.nlm.nih.gov/Blast.cgi).

### Statistical Analysis

The presence of statistical differences in the frequency of HEV and pAdV detection in liver and blood samples was assessed by Student’s *t*-test for independent pairs with previously-tested homogeneity of variance using the F test. Analysis of variance with confidence intervals (LSD) was used to assess the differences in frequency in occurrence of the viruses in tissues (liver and blood samples) of slaughtered animals originating from small, medium and large farms. Calculations were made with Statgraphics Centurion version XV (Statgraphics Technologies, USA).

## Results

### Virus Detection in Blood and Liver

HEV RNA was found in 6 (2.4%; 95% CI: 0.9–5.2%) out of 246 samples of tested foodstuffs. All blood samples showed a 100% (very good) extraction efficiency. In the case of livers the mean extraction efficiency was good (24.07  ±  30.15%) with values ranging from 1.1 to 100%. None of the samples showed poor virus recovery. Virus was detected in five samples of pig’s blood (3.4%; 95% CI: 1.1–7.8%) and in only one liver sample (1.0%; 95% CI: 0.0–5.0%) (Table 2). There were no significant differences observed in the frequency of HEV occurrence between blood and liver samples (*t* = 1.33; *p* = 0.182 > 0.05). The viral load in blood ranged from 11 G.C./ml to 9.0 × 10^4^ G.C./ml, whereas in liver it was as low as 16 G.C./g. PAdV was detected more frequently in pig blood than in liver (*t* = 4.65; *p* = 0.000 < 0.05) with an overall 13% (95% CI: 8.0–19.5%) prevalence among the tested samples (Table 2). Only finishers raised on large farms were HEV-positive: all 5 pooled blood samples (4.5%; 95% CI: 1.4–10.2%) containing viral RNA came from among the 111 such operations (Table 3). The geographical distribution of the sampled farms from which pooled blood yielded HEV was Mazovia, with two samples; Kujawy-Pomerania, with two samples; and Podlaskie with one sample. PAdV was detected in pigs regardless of the farm size from which they originated. Consequently, there were 2 (14.3; CI 1.7–42.8) pAdV-positive samples of pooled blood taken from animals representing medium-sized farms, 4 (19.0; CI 5.4–41.9) from small and 13 (11.7; CI 6.3–19.2) from large farms (Table 3). The number of animals raised on the farm (the farm size) had no influence on the occurrence of HEV or pAdV infections in pigs (*F* = 0.81, *p* = 0.447 > 0.05 for HEV; *F* = 0.42, *p* = 0.655 > 0.05 for pAdV).

**Table 2:** Results of detection of HEV and pAdV in pig’s blood and livers

Sample type	No. of samples	HEV			pAdV	
		No. of positive samples (%; CI 95%)	Cq ± SD	Virus load (G.C./ml or g)	No. of positive samples (%; CI 95%)	Cq ± SD
Blood	146	5 (3.4; 1.1–7.8)	36.0 ± 2.5	11 – 9.0 × 10^4^	19 (13.0; 8.0–19.5)	39.0 ± 1.3
Liver	100	1 (1.0; 0.0–5.0)	40.7	16	0 (0; 0–3.6)	–

**Table 3:** Prevalence of HEV and pAdV in pig’s blood in relation to the size of the farm breeding the animals

Farm size	No. of farms	No. of pooled blood samples*	No. of positive samples	
			HEV (%; CI 95%)	pAdV (%; CI 95%)
Small	21	21	0 (0; CI 0–16.1)	4 (19.0; CI 5.4–41.9)
Medium	7	14	0 (0; CI 0–23.1)	2 (14.3; CI 1.7–42.8)
Large	37	111	5 (4.5; CI 1.4–10.2)	13 (11.7; CI 6.3–19.2)
Total		146	5 (3.4; CI 1.1–7.8)	19 (13.0; CI 8.0–19.5)

*Each small-scale farm was represented by one pooled blood sample (each pooled sample consisted of blood taken from 10 pigs), the medium-scale and large-scale size farms were represented by two and three samples respectively

A specific PCR product corresponding to the HEV ORF 2 region was obtained for five virus strains. Four strains belonged to the 3f virus subtype (previously designated 3 l (p) subtype, EU360977, bootstrap value >84%), and one to the 3e subtype (previously undesignated, AB248521, bootstrap value 91%) (Smith et al., 2020). HEV ORF2 sequences were deposited in GenBank under the accession numbers MT773598–MT773600 and MT780502. Nucleotide sequences of the hexon capsid protein of all pAdV strains belonged to serotype 5 (98.5% sequence similarity to pAdV-5 reference sequence AF289262) with a mutual 100% sequence similarity. A representative sequence of this pAdV-5 strain was submitted to GenBank under the accession number MT895403.

## Discussion

In this study, the presence of HEV RNA was only detected in 5 (3.4%) out of 146 samples of pig’s blood and in one liver, which constituted a mere 1% of the tested liver samples. There were no statistically significant differences observed between the virus’ occurrence in liver samples and blood samples. HEV has also been detected in livers of locally raised pigs in Africa (de Paula et al., 2013; Temmam et al., 2013), albeit occasionally; in contrast in Europe up to 31% of retailed liver contained the virus (Feurer et al., 2018; Forgách et al., 2010). However, there were differences observed between countries. For example, 15.5% of pig livers collected at the time of animal slaughter were positive for HEV RNA (Garcia et al., 2020). Nearly three times lower (6.3%) frequency of virus detection in slaughtered pigs was reported by Geng et al. (2019) with not significant difference in detection rates between livers collected from slaughterhouses and retail markets. When a pork production chain was analysed in the Czech Republic, Italy and Spain, a mean 4% prevalence of HEV was detected in livers collected at a slaughterhouse stage (Di Bartolo et al., 2012). Compared to the European food market, HEV was detected in pig’s livers with a lower frequency in Asia (0.28–12.4%) (Hoan et al., 2019; Intharasongkroh et al., 2016) and in North and South America (4.9–11%) (Feagins et al., 2007; Wilhelm et al., 2014). As with pig’s livers, viral RNA has also been sporadically (1.8%) found in pig’s blood, (Geng et al., 2019), and sera (6.7%) of slaughtered animals (Garcia et al. 2020), despite the virus being highly prevalent (23.4–100%) in blood and blood-derived by-products such as haemoglobin, fibrinogen and plasma (Boxman et al., 2017; Geng et al., 2019). Differences in virus occurrence in edible blood between studies may stem from differences observed between countries in natural virus prevalence in pigs (Berto, Backer, et al., 2012; Di Bartolo et al., 2008; Lee et al., 2020) and the age of slaughtered animals, as the likelihood of viraemia increases in younger pigs (Meng et al., 1997; Sooryanarain et al., 2020; Wu et al., 2002).

In this study HEV was more frequently detected in blood that in livers. Its sporadic detection in pork slaughter by-products could to some extent reflect a low natural prevalence of the virus in animals at the time of slaughter. Additionally, samples of blood and liver were not collected at the same stage of the pork production chain, therefore different detection rate of HEV RNA in tested samples could be expected. Furthermore, they were also originated from different animals kept on distinct farms at different geographical locations, where HEV infections in pigs may not be frequently prevalent. This randomized sampling resulted in getting an averaged but reliable results of the HEV occurrence in tested slaughter by-products. Likewise, the discrepancy in virus detection rate between samples was not influenced by the detection method used as it was confirmed by good SPCV recovery from tested samples along with the absence of inhibition of molecular reactions.

Blood sausage and black pudding are the most popular processed offal products containing pig’s blood in Europe. Pig’s blood is also added to many national dishes, for example, pig’s organ soup (Malaysia and Singapore), dinuguan (Philippines), mykyrokka (Finland), tiết canh (Vietnam) or svartsoppa in Sweden. Besides whole blood, its fractions, i.e. sediment of red blood cells and plasma are also used in the meat industry (Banach & Makara, 2011; Pełczyńska & Libelt, 1999). Pig’s liver is marketed to the consumer raw as a separate ingredient or is added to the raw or dried liver sausages sold in Europe (Colson et al., 2010; Giannini et al., 2018; Renou, 2014).

Generally meat products undergo heat treatment before consumption; nevertheless there are still some food varieties containing blood or liver which are consumed raw or subjected to thermal processing which might be insufficient to inactivate the virus (Barnaud et al., 2012; Pavio et al., 2014), whereby a food-related risk of HEV infection is likely to arise (Boxman et al., 2019; Giannini et al., 2018; Pavio et al., 2014). For example, HEV RNA has been detected in pork liver sausages sold in the UK (9.5%) (Berto, Martelli, et al., 2012), Spain (6%) (Di Bartolo et al., 2012), Italy (22.2%) (Di Bartolo et al., 2015), France (29–30%) (Pavio et al., 2014), and Germany (26%) (Szabo et al., 2015). The virus has been found with a higher frequency (> 36%) in pork pâté and blood sausages (Heldt et al., 2016; Mykytczuk et al., 2017).

It is noteworthy that HEV strains detected in this study were identified as gt 3e and 3f subtypes. Our results are therefore consistent with previous findings in which virus strains of the same subtypes have been detected in pigs liver (Bouwknegt et al., 2007; Bouquet et al., 2011; Garcia et al., 2020), ready-to-eat food containing pigs liver (Colson et al., 2010) or other type of pork meat produce (Di Bartolo et al., 2012).

The role of these strains in foodborne HEV transmission was evidenced by the detection of the autochthonous cases of human infection in France (Bouquet et al., 2011). Nevertheless, detection of HEV RNA does not itself indicate whether infectious or non-infectious virus particles are present (Althof et al., 2019; Szabo et al., 2015). In this study, an attempt to determine the infectivity of the HEV strains detected in raw food material was not undertaken due to an insufficient number of virus particles (< 10^4^ G.C./ml) contaminating food items (Takahashi et al., 2012) and the lack of a cell culture system efficiently propagating wild-type virus strains (Schemmerer et al., 2019).

Among the risk factors determining the occurrence of HEV infections in pigs are the farm size and farming practices (Di Bartolo et al., 2008; Hinjoy et al., 2013; Jinshan et al., 2010; Li et al., 2009; Rutjes et al., 2014). A higher seroprevalence rate has been observed among pigs from medium-sized farms raising 30–300 animals compared to large and small farms (Hinjoy et al., 2013). It could have resulted from poorer environmental hygiene conditions on medium-sized farms and from differences in feeding and farm management. It has been shown that the higher frequency of HEV infections in pigs may be influenced by more than poor on-farm hygienic conditions, being also possibly caused by a later weaning age of piglets, animal housing and separation (Walachowski et al., 2014).

In this study, there were no differences observed in the frequency of virus occurrence in animal tissues which originated from herds which were different in size and geographical location. Also, the animal diet based on commercial feed or feed made on the farm and which floor system (slatted floor or straw bedding) was used had no significant impact on the frequency of infections in slaughtered animals. HEV detection being only sporadic in slaughtered pigs could mainly be associated with animal age: active infection mostly occurs in young animals, whereas the majority of pigs at the time of slaughter are virus-free (Dalton et al., 2019; Feagins et al., 2007). In addition, the similarity in virus prevalence between the farms might have resulted from strict compliance with the biosecurity measures introduced on farms to stop the spread of african swine fever virus (ASFV) in Poland. It is believed that as high a proportion as 92.8% of slaughtered animals have had contact with the virus (Grierson et al., 2015); however, viraemia was only observed in a small percentage of slaughtered pigs (de Deus et al., 2008; Grierson et al., 2015; Sooryanarain et al., 2020). The detection of HEV RNA may be associated with infection occurring close to slaughter time, if superspreaders with external faecal shedding were present in the herd (Grierson et al., 2015).

Information on the possible occurrence of enteric viruses in food can also be gained through detection of viral indicators of faecal contamination. PAdV is abundantly shed in pig faeces; therefore it has been used as an indicator for source tracking of animal faecal contamination in meat and fresh produce supply chains (Jones & Muehlhauser, 2017; Kokkinos et al., 2017; Maunula et al., 2013; Paszkiewicz et al., 2016). In this study, corroboration for the occurrence of HEV in pig’s blood and livers being sporadic also comes from the number of samples tested positive for pAdV being low. Virus was only found in blood and none of the tested livers yielded positive results. To reveal the origin of the detected virus strains, sequencing of pAdV- positive amplicons was undertaken. All sequenced strains were identified as pADV-C (serotype 5) which are associated with respiratory swine disease (Horak & Leedom Larson, 2016). It is highly likely that respiratory infection may have more general course with viremia occurring more frequently. This could explain a frequent pAdV detection in pig’s blood then in liver. This finding indicates that the use of pAdVs as an indicator of fecal contamination may have a limited value when an identification of virus serotype is not performed. On the other hand, regardless the detected pAdV serotype, and given the similarities in the frequency of HEV and pAdV occurrence in tissues of slaughtered animals, it could be assumed that the infection prevalence caused by these viruses will be similar and that the virus burden in the pig population in Poland is likely to be small. This may be partly explained by the effectiveness of biosafety measures introduced on the Polish farms to combat ASFV infections, which may have also significantly reduced the transmission of other pig viruses including HEV.

This study has two possible limitations: (i) related to the detection methodology used and (ii) an underestimation of the results of virus occurrence in pig’s blood tested as pooled samples. The suitability of the silica-membrane-based RNA extraction method for extraction of HEV RNA from pork slaughter by-products such as pigs blood (Chelli et al., 2021; Crossan et al., 2015; Garcia et al., 2020; Thiry et al., 2014) and livers (Bouwknegt et al., 2007; Bouquet et al., 2011; Di Bartolo et al., 2012; Garcia et al., 2020) has previously been shown. The mechanical tissue disruption performed jointly with a chemical cell lysis enabled an efficient isolation of nucleic acids, as the mean value of SPCV recovery obtained for the tested samples was equal to (69.14  ±  42.0%). It was comparable to the recovery rate of MNV-1 (50.3 ± 1.83%) which served as SPCV for blood, stool, muscle and samples of internal organs collected from slaughtered pigs (Garcia et al., 2020). A sporadic detection of the virus in blood and livers may have resulted from its low natural prevalence in animals at the time of slaughter, rather than from limitations of the method. Although the sample size used for analysis is crucial for a successful virus detection that higher amount of co-extracted food-related inhibitory substances could lower sensitivity of HEV detection (Szabo et al., 2015). Nevertheless, the use of spin-column based nucleic acid extraction and purification method allowed to remove or significantly reduce an inhibitory effect of the sample improving the efficiency of nucleic acid extraction. Likewise, as previously demonstrated the assay targeting virus ORF2/3 region showed the best detection performance for HEV RNA in plasma and human blood samples. This assay in conjunction with the membrane-based RNA extraction was able to detect HEV gt3 in human blood at the concentration of 2500Ul/ml (Mokhtari et al., 2013). In this study the same method employing the amplification enzyme-mix and a detection platform, however with a higher volume of sample RNA was used for virus detection in pig’s blood. Nevertheless, that change seems to be beneficial for the method as it could only increase its sensitivity. Therefore, it is conceivable that method’s LOD for testing of pig’s blood could be at least similar as for samples of human blood.

A possible underestimation of the results of virus occurrence when pooled blood samples are tested mainly refers to samples which could contain the virus at the concentration equal to or slightly above the detection limit of the method. It relates to the dilution effect of the positive sample by other uncontaminated samples being in the pool. In food virology testing of pooled samples is commonly used, for example for shellfish testing (Rzeżutka & Carducci, 2013) and this complies to the recommendations of an ISO standard for virus detection in food (Anonymous, 2019). In the case of edible blood and its by-products they have previously been successfully tested for HEV as pooled samples (Boxman et al., 2017). It is also important to emphasize that compared to studies of Boxman et al. (2017) in this study a tested portion of blood sample was representing a much higher volume of individual sample being in the pool. In contrary to food samples but similarly to other clinical specimens, viruses are homogenously distributed in blood. This feature significantly reduces the risk of getting the false-negative results when blood is tested as pooled sample.

## Conclusions

This study provides valuable data on the occurrence of HEV in pig’s blood and retailed pig’s livers in Poland. There were no differences observed in the frequency of virus prevalence in slaughtered pigs correlating with farm size (number of animals bred) or specific farm husbandry and feeding practices. Although the virus was only sporadically detected, its occurrence still remains consequential for consumers as it demonstrates that HEV-contaminated pig tissues can enter the food chain. Of note is that they are the major food ingredients for production of the variety of edible offal meat products which may not undergo sufficient heat-treatment (if any) to inactivate the virus (Colson et al., 2010; Giannini et al., 2018; Renou, 2014). Although meat products containing blood and/or pork liver are very popular with Polish consumers, it is difficult to unequivocally show that their consumption caused any foodborne infection or food-related intoxication outbreaks. Detection of HEV in food chain–bound pig tissues supports the assumption that the risk of HEV infection related to consumption of ready-to-eat offal-derived foodstuffs exists, however it should be verified by subsequent food testing.

## Data Availability

Sequences determined in this study were submitted to GenBank database under the Accession Number MT773598 – MT773600, MT780502 and MT895403.
